# Supplementary figures and images for: Stromule extension along microtubules coordinated with actin-mediated anchoring guides perinuclear chloroplast movement during innate immunity
Source: eLife. 2018 Jan 17;7:e23625. doi: 10.7554/eLife.23625 (PMC5815851; doi:10.7554/eLife.23625)

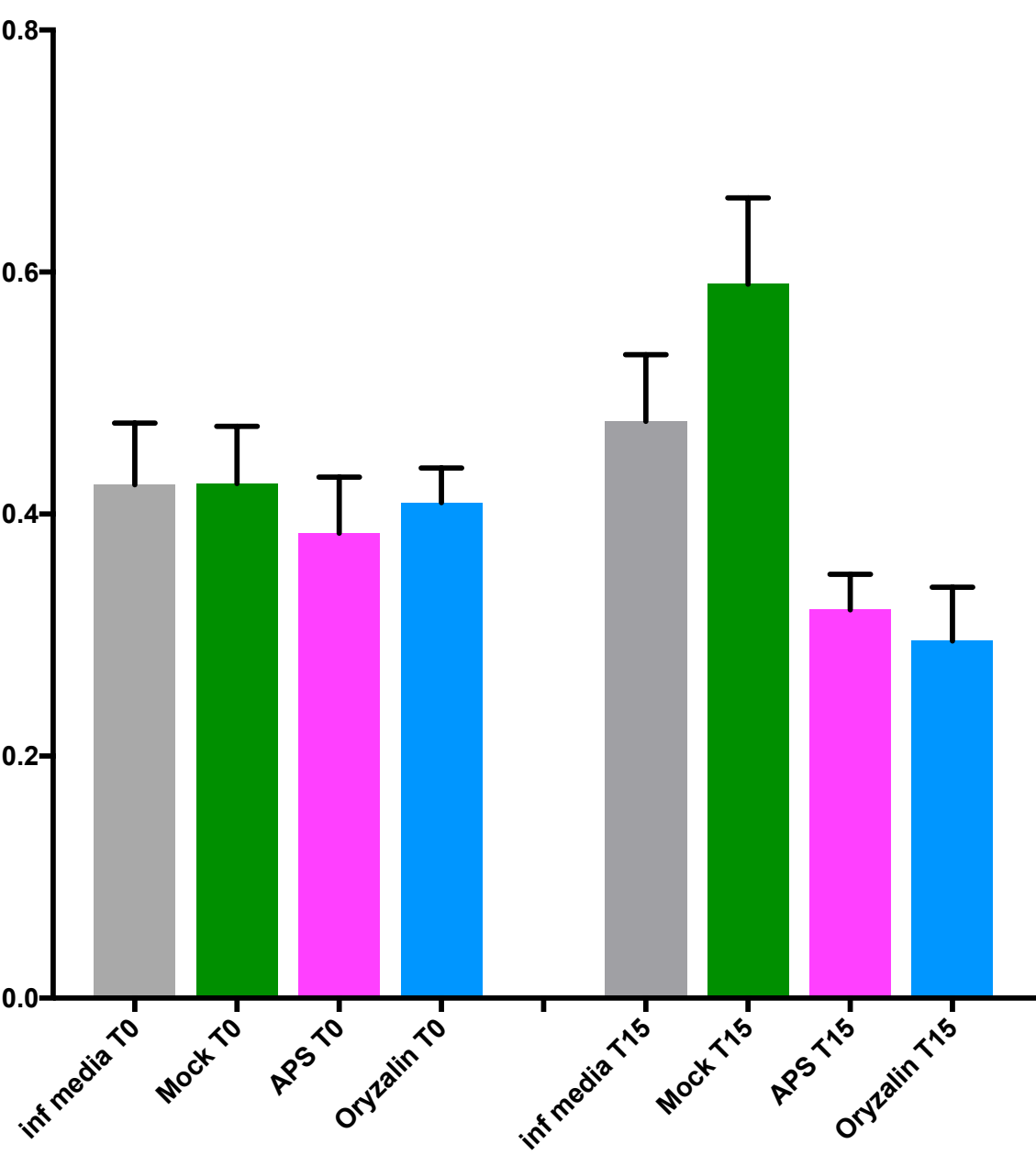

Supplement: Figure 3—source data 1. — Raw datapoints of stromule frequency after APM, Oryzalin, or Paclitaxel, presented in Figure 3B and D. Statistical analyses and the original graph generated by Prism7 were also included. [file elife-23625-fig3-data1.zip › Figure 3-source file 1/APS and Oryzalin.pdf]

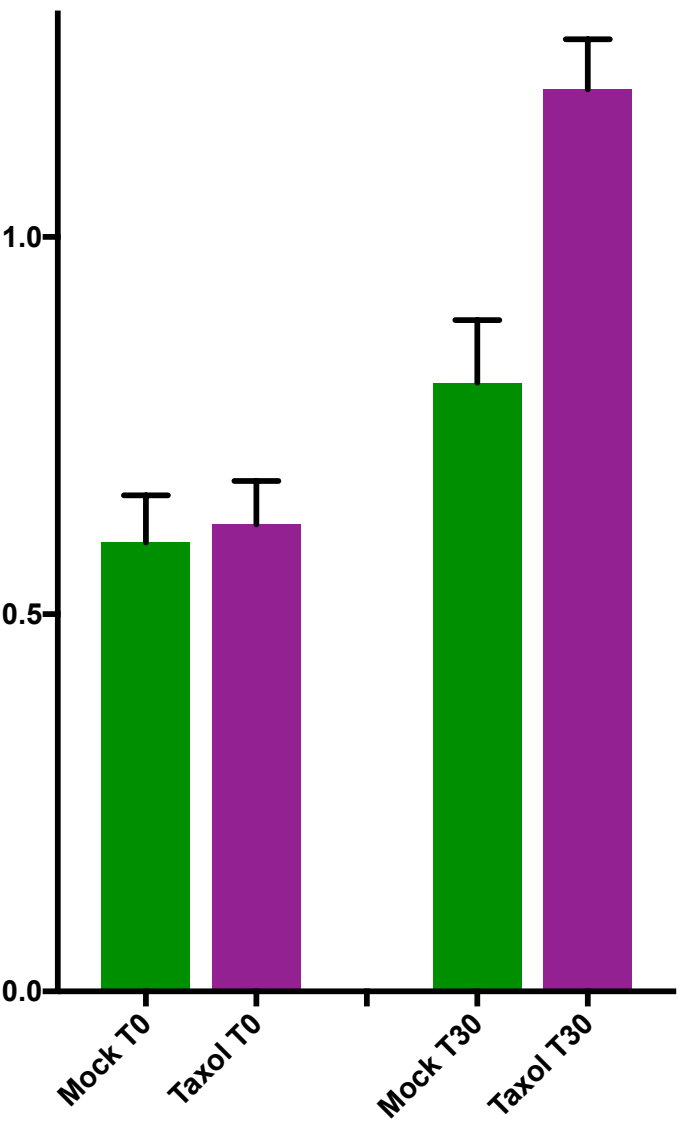

Supplement: Figure 3—source data 1. — Raw datapoints of stromule frequency after APM, Oryzalin, or Paclitaxel, presented in Figure 3B and D. Statistical analyses and the original graph generated by Prism7 were also included. [file elife-23625-fig3-data1.zip › Figure 3-source file 1/Taxol.pdf]

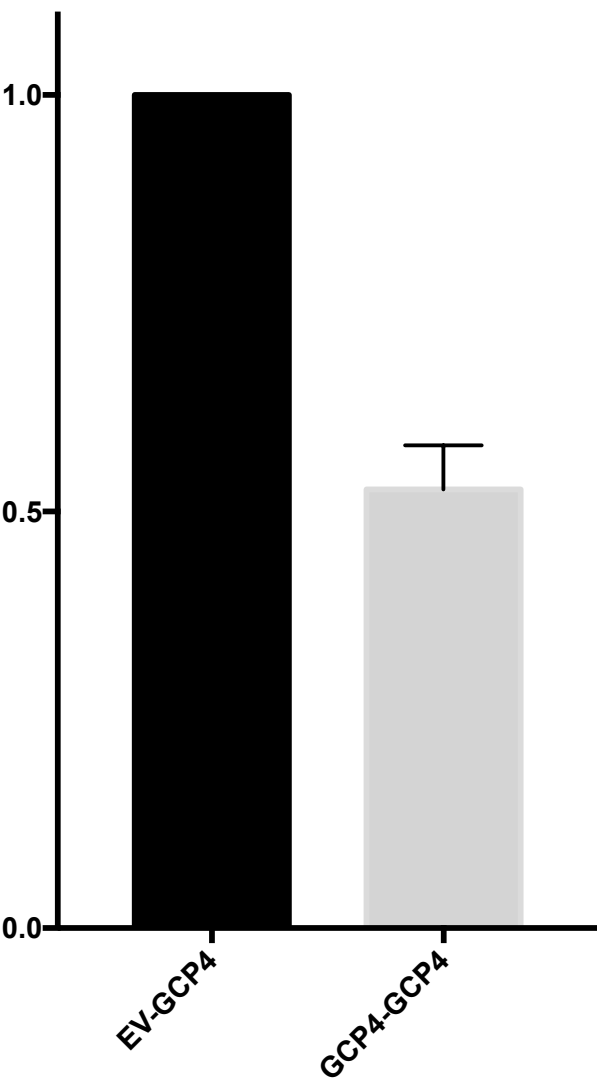

Supplement: Figure 4—source data 1. — Statistical analyses and the graph of the quantitative RT-PCR for the NbGCP4 gene silencing presented in Figure 4B were generated by Prism7. [file elife-23625-fig4-data1.zip › Figure 4-source file 1/Q-RT-PCR result.pdf]

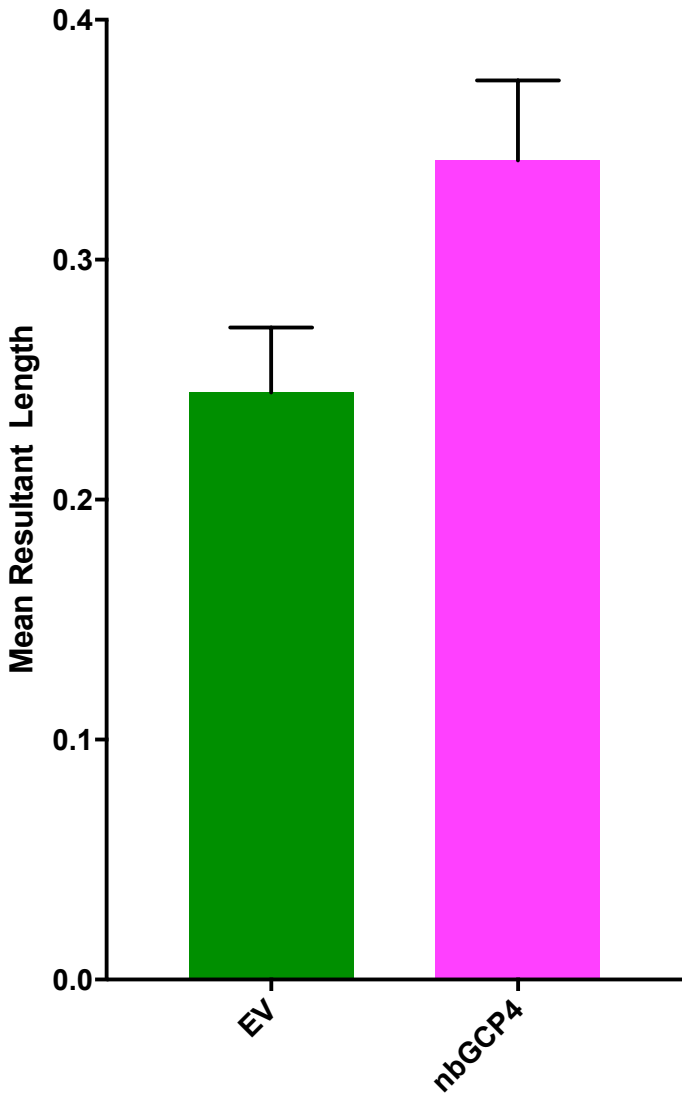

Supplement: Figure 4—source data 2. — Statistical analyses and the graph of the Azimuthal angles of the MT in the NbGCP4 silenced plants presented in Figure 4C were generated by Prism7. [file elife-23625-fig4-data2.zip › Figure 4-source file 2/MRL Azimuthal.pdf]

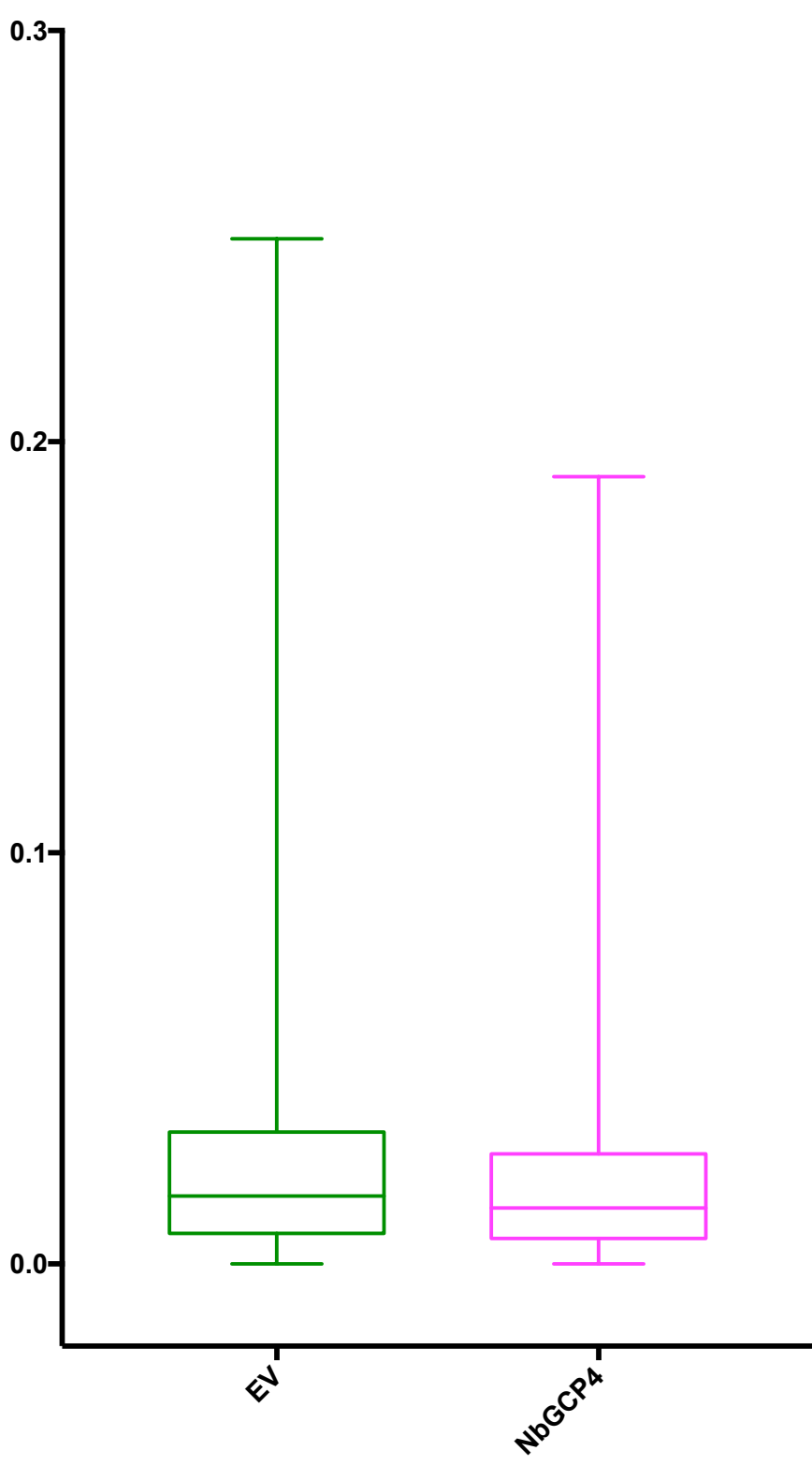

Supplement: Figure 4—source data 3. — Statistical analyses and the graph of the curvature of the MT in the NbGCP4 silenced plants presented in Figure 4E were generated by Prism7. [file elife-23625-fig4-data3.zip › Figure 4-source file 3/all 5 rep combined to one column.pdf]

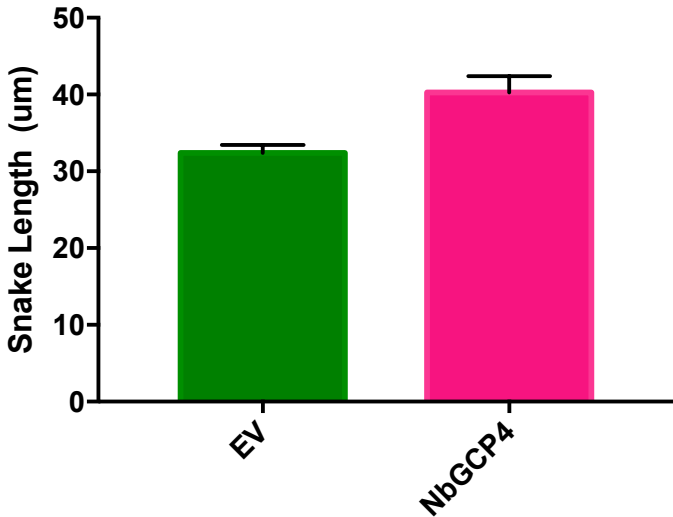

Supplement: Figure 4—source data 4. — Statistical analyses and the graph of the SOAX analysis of the MT in the NbGCP4 silenced plants presented in Figure 4F were generated by Prism7. [file elife-23625-fig4-data4.zip › Figure 4-source file 4/Final graph.pdf]

# stromule velocity

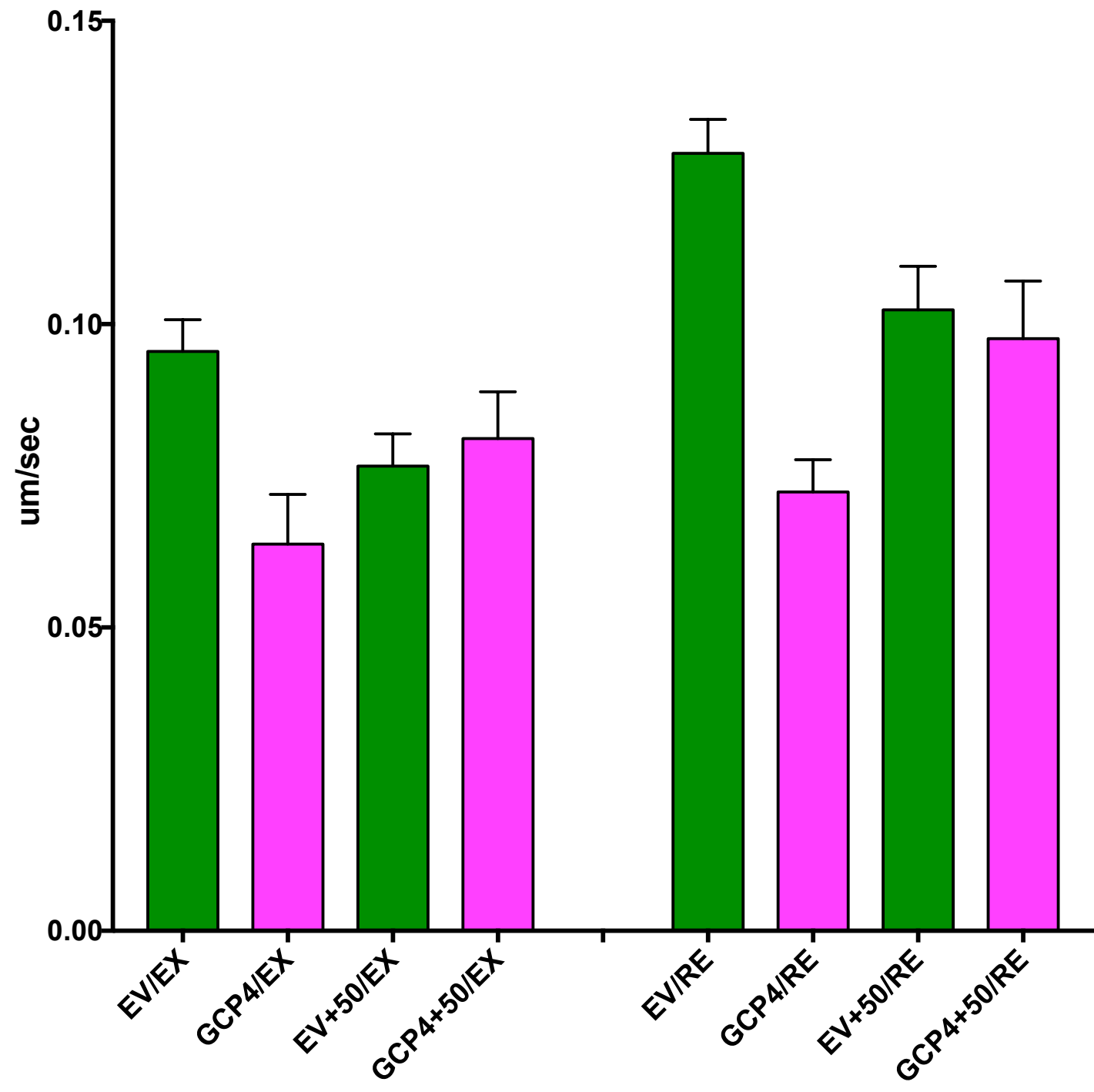

Supplement: Figure 5—source data 1. — Statistical analyses and the graph of the stromule frequency, length, and velocity in the NbGCP4 silenced plants presented in Figure 5B-D by Prism7 are included. [file elife-23625-fig5-data1.zip › Figure 5-source file 2/stromule velocities.pdf]

# 710 Azimuthal MRL

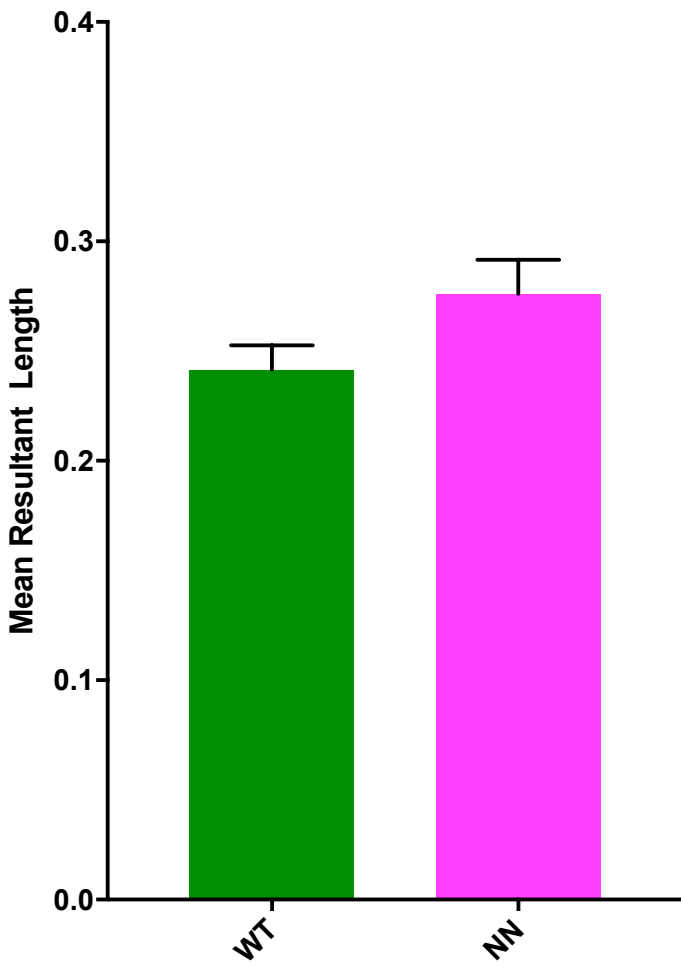

Supplement: Figure 5—source data 2. — Statistical analyses and the graph of the MT distribution presented in Figure 5F-H were generated by Prism7. [file elife-23625-fig5-data2.zip › Figure 5-source file 3/710 Azimuthal MRL.pdf]

## 710 Curvature

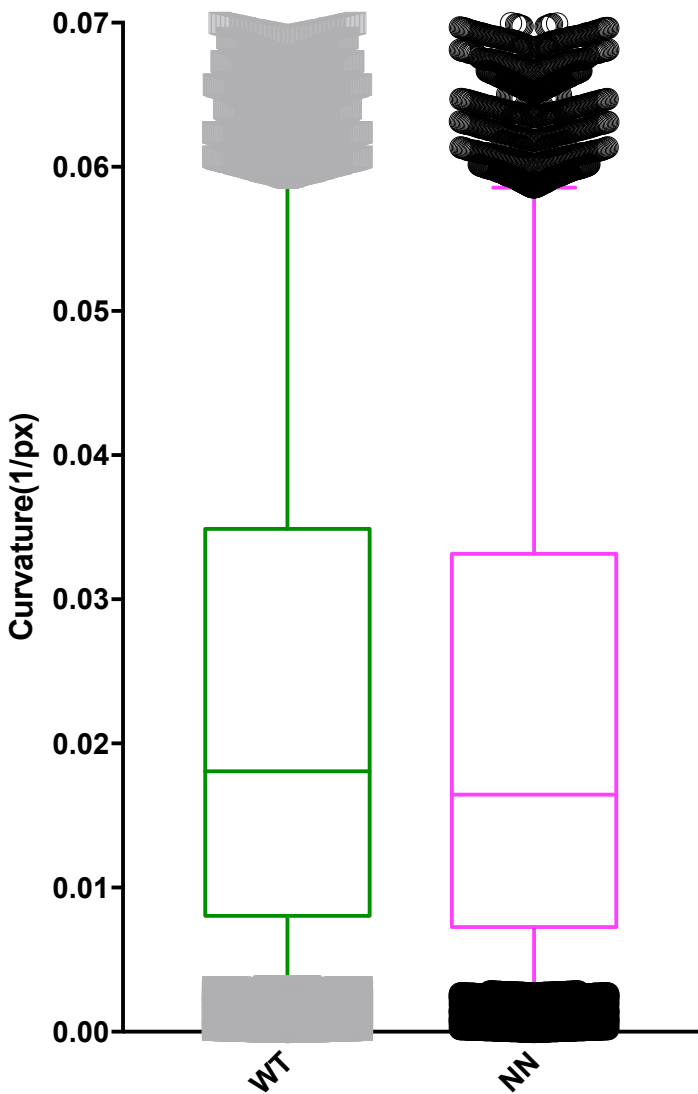

Supplement: Figure 5—source data 2. — Statistical analyses and the graph of the MT distribution presented in Figure 5F-H were generated by Prism7. [file elife-23625-fig5-data2.zip › Figure 5-source file 3/710 Curvature.pdf]

## 710 Length

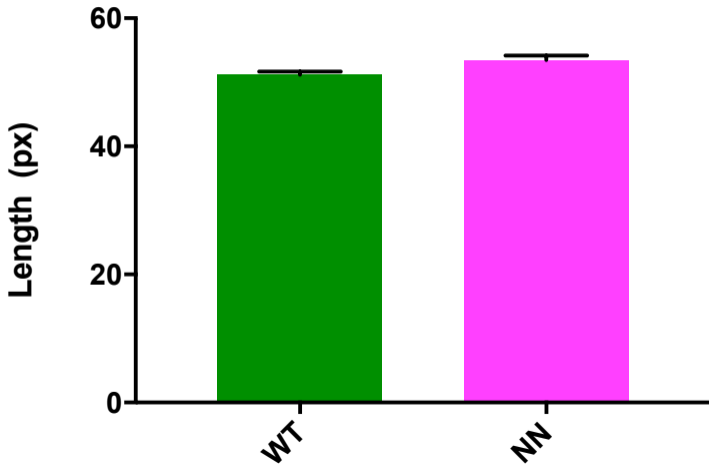

Supplement: Figure 5—source data 2. — Statistical analyses and the graph of the MT distribution presented in Figure 5F-H were generated by Prism7. [file elife-23625-fig5-data2.zip › Figure 5-source file 3/710 Length.pdf]

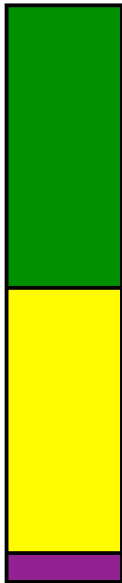

49.00% constant

46.00% sudden

5.00% side

Supplement: Figure 5—figure supplement 1—source data 1. — The statistics of quantitative analysis of stromule length and movement in VIGS-NbGCP4 plants by Prism7 is included the raw data points, statistical analyses, as well as the original graphs of Figure 5—figure supplement 1A and B. [file elife-23625-fig5-figsupp1-data1.zip › Figure 5-figure supplement 1-source file 1/Data 8 [VIGS-EV+p50].pdf]

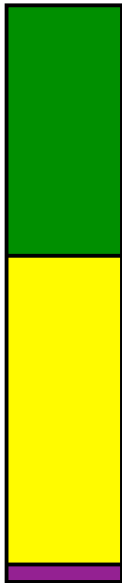

43.43% constant

53.54% sudden

3.03% side

Supplement: Figure 5—figure supplement 1—source data 1. — The statistics of quantitative analysis of stromule length and movement in VIGS-NbGCP4 plants by Prism7 is included the raw data points, statistical analyses, as well as the original graphs of Figure 5—figure supplement 1A and B. [file elife-23625-fig5-figsupp1-data1.zip › Figure 5-figure supplement 1-source file 1/Data 8 [VIGS-EV].pdf]

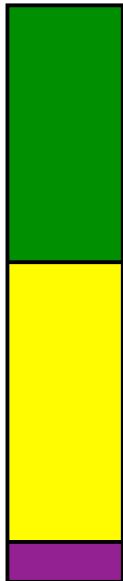

44.55% constant

48.51% sudden

6.93% side

Supplement: Figure 5—figure supplement 1—source data 1. — The statistics of quantitative analysis of stromule length and movement in VIGS-NbGCP4 plants by Prism7 is included the raw data points, statistical analyses, as well as the original graphs of Figure 5—figure supplement 1A and B. [file elife-23625-fig5-figsupp1-data1.zip › Figure 5-figure supplement 1-source file 1/Data 8 [VIGS-GCP4 +p50].pdf]

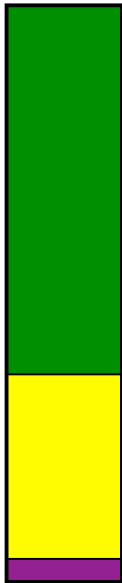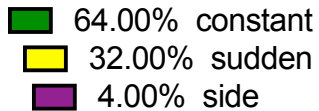

Supplement: Figure 5—figure supplement 1—source data 1. — The statistics of quantitative analysis of stromule length and movement in VIGS-NbGCP4 plants by Prism7 is included the raw data points, statistical analyses, as well as the original graphs of Figure 5—figure supplement 1A and B. [file elife-23625-fig5-figsupp1-data1.zip › Figure 5-figure supplement 1-source file 1/Data 8 [VIGS-GCP4].pdf]

**EV+50**

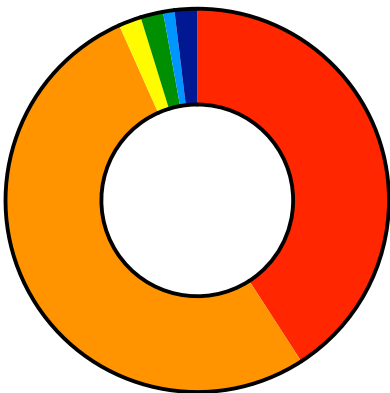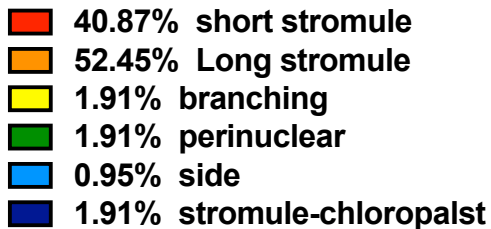

**Total=1.04857**

Supplement: Figure 5—figure supplement 1—source data 1. — The statistics of quantitative analysis of stromule length and movement in VIGS-NbGCP4 plants by Prism7 is included the raw data points, statistical analyses, as well as the original graphs of Figure 5—figure supplement 1A and B. [file elife-23625-fig5-figsupp1-data1.zip › Figure 5-figure supplement 1-source file 1/stromule length [EV+50].pdf]

**EV**

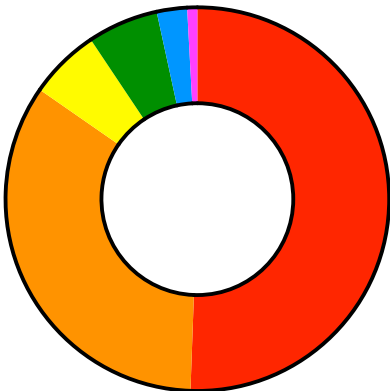

- 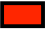 **50.57% short stromule**
- 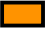 **34.09% Long stromule**
- 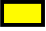 **5.97% branching**
- 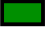 **5.97% perinuclear**
- 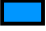 **2.56% side**
- 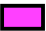 **0.85% stromule-stromule**

**Total=1.17344**

Supplement: Figure 5—figure supplement 1—source data 1. — The statistics of quantitative analysis of stromule length and movement in VIGS-NbGCP4 plants by Prism7 is included the raw data points, statistical analyses, as well as the original graphs of Figure 5—figure supplement 1A and B. [file elife-23625-fig5-figsupp1-data1.zip › Figure 5-figure supplement 1-source file 1/stromule length [EV].pdf]

## GCP4+50

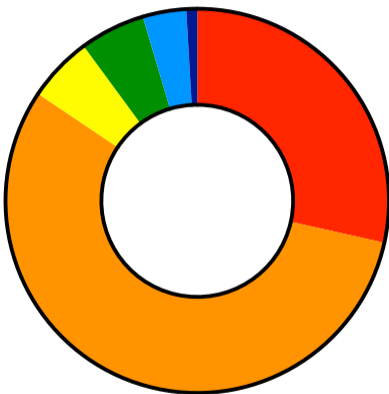

- 28.50% short stromule
- 55.91% Long stromule
- 5.50% branching
- 5.50% perinuclear
- 3.67% side
- 0.92% stromule-chloropalst

Total=1.09032

Supplement: Figure 5—figure supplement 1—source data 1. — The statistics of quantitative analysis of stromule length and movement in VIGS-NbGCP4 plants by Prism7 is included the raw data points, statistical analyses, as well as the original graphs of Figure 5—figure supplement 1A and B. [file elife-23625-fig5-figsupp1-data1.zip › Figure 5-figure supplement 1-source file 1/stromule length [GCP4+50].pdf]

43.88% short stromule

56.12% long stromule

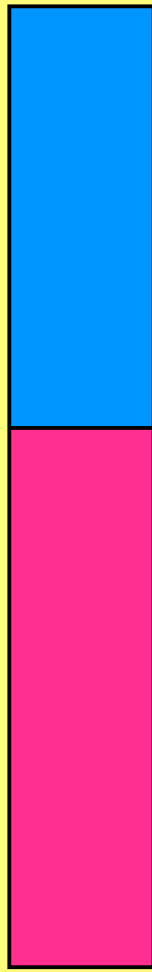

Supplement: Figure 5—figure supplement 1—source data 1. — The statistics of quantitative analysis of stromule length and movement in VIGS-NbGCP4 plants by Prism7 is included the raw data points, statistical analyses, as well as the original graphs of Figure 5—figure supplement 1A and B. [file elife-23625-fig5-figsupp1-data1.zip › Figure 5-figure supplement 1-source file 1/stromule length with new data [VIGS-EV+p50].pdf]

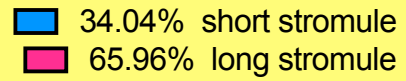

Supplement: Figure 5—figure supplement 1—source data 1. — The statistics of quantitative analysis of stromule length and movement in VIGS-NbGCP4 plants by Prism7 is included the raw data points, statistical analyses, as well as the original graphs of Figure 5—figure supplement 1A and B. [file elife-23625-fig5-figsupp1-data1.zip › Figure 5-figure supplement 1-source file 1/stromule length with new data [VIGS-GCP4 +p50].pdf]

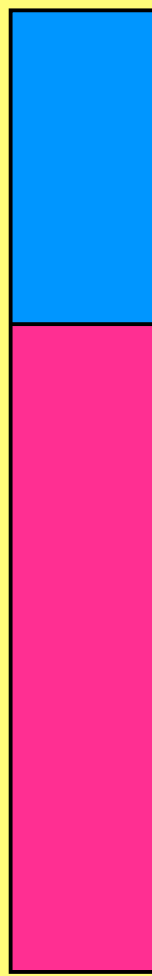

32.63% short stromule

67.37% long stromule

Supplement: Figure 5—figure supplement 1—source data 1. — The statistics of quantitative analysis of stromule length and movement in VIGS-NbGCP4 plants by Prism7 is included the raw data points, statistical analyses, as well as the original graphs of Figure 5—figure supplement 1A and B. [file elife-23625-fig5-figsupp1-data1.zip › Figure 5-figure supplement 1-source file 1/stromule length with new data [VIGS-GCP4].pdf]

**EV+50**

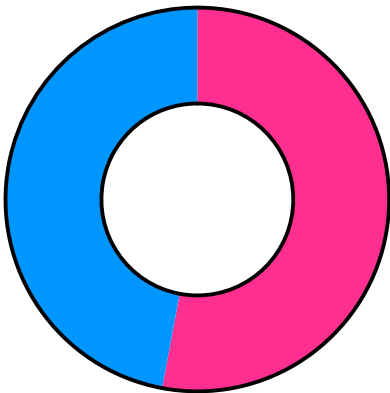

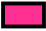 **52.94% Fast movement**  
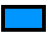 **47.06% Slow movement**

**Total=0.85**

Supplement: Figure 5—figure supplement 1—source data 1. — The statistics of quantitative analysis of stromule length and movement in VIGS-NbGCP4 plants by Prism7 is included the raw data points, statistical analyses, as well as the original graphs of Figure 5—figure supplement 1A and B. [file elife-23625-fig5-figsupp1-data1.zip › Figure 5-figure supplement 1-source file 1/stromule movement [EV+50].pdf]

**EV**

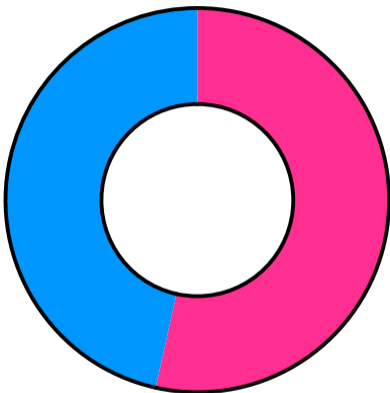

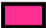 **53.49% Fast movement**  
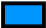 **46.51% Slow movement**

**Total=0.86**

Supplement: Figure 5—figure supplement 1—source data 1. — The statistics of quantitative analysis of stromule length and movement in VIGS-NbGCP4 plants by Prism7 is included the raw data points, statistical analyses, as well as the original graphs of Figure 5—figure supplement 1A and B. [file elife-23625-fig5-figsupp1-data1.zip › Figure 5-figure supplement 1-source file 1/stromule movement [EV].pdf]

**GCP4+50**

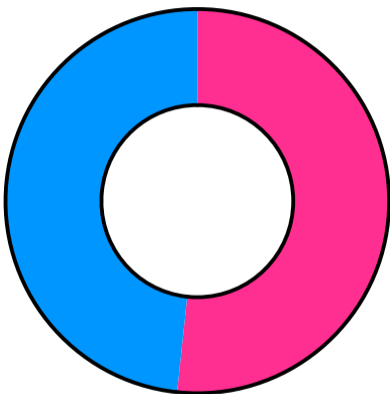

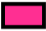 **51.69% Fast movement**  
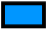 **48.31% Slow movement**

**Total=0.89**

Supplement: Figure 5—figure supplement 1—source data 1. — The statistics of quantitative analysis of stromule length and movement in VIGS-NbGCP4 plants by Prism7 is included the raw data points, statistical analyses, as well as the original graphs of Figure 5—figure supplement 1A and B. [file elife-23625-fig5-figsupp1-data1.zip › Figure 5-figure supplement 1-source file 1/stromule movement [GCP4+50].pdf]

**GCP4**

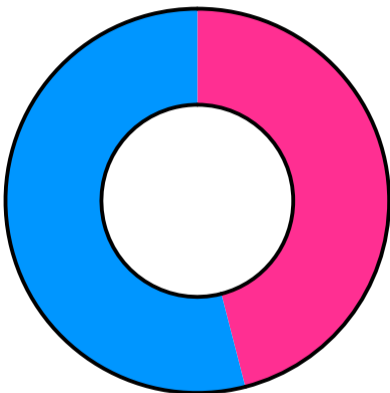

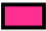 **45.98% Fast movement**  
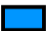 **54.02% Slow movement**

**Total=0.87**

Supplement: Figure 5—figure supplement 1—source data 1. — The statistics of quantitative analysis of stromule length and movement in VIGS-NbGCP4 plants by Prism7 is included the raw data points, statistical analyses, as well as the original graphs of Figure 5—figure supplement 1A and B. [file elife-23625-fig5-figsupp1-data1.zip › Figure 5-figure supplement 1-source file 1/stromule movement [GCP4].pdf]

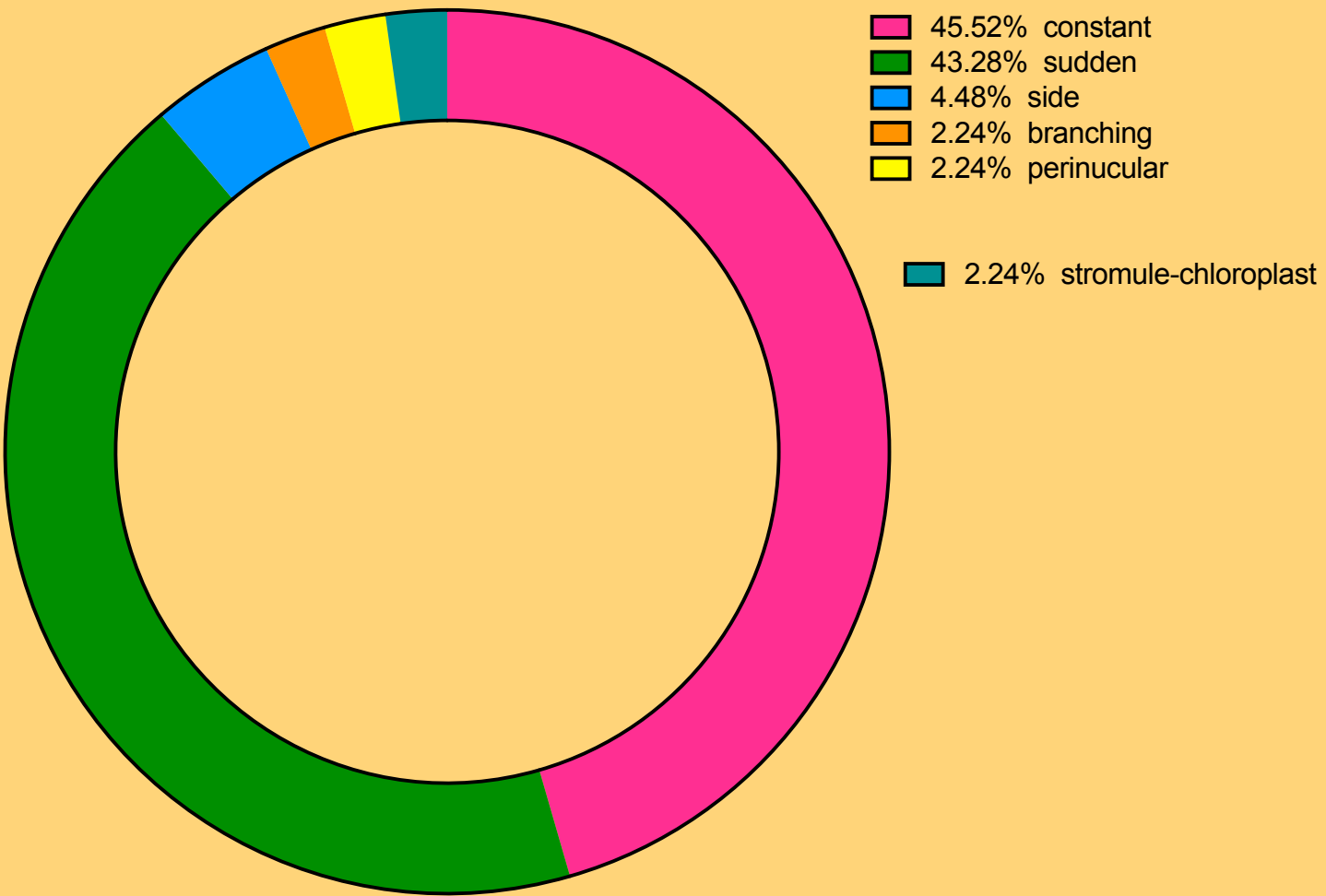

Total=134

Supplement: Figure 5—figure supplement 1—source data 1. — The statistics of quantitative analysis of stromule length and movement in VIGS-NbGCP4 plants by Prism7 is included the raw data points, statistical analyses, as well as the original graphs of Figure 5—figure supplement 1A and B. [file elife-23625-fig5-figsupp1-data1.zip › Figure 5-figure supplement 1-source file 1/stromule movement with new data [VIGS-EV+p50].pdf]

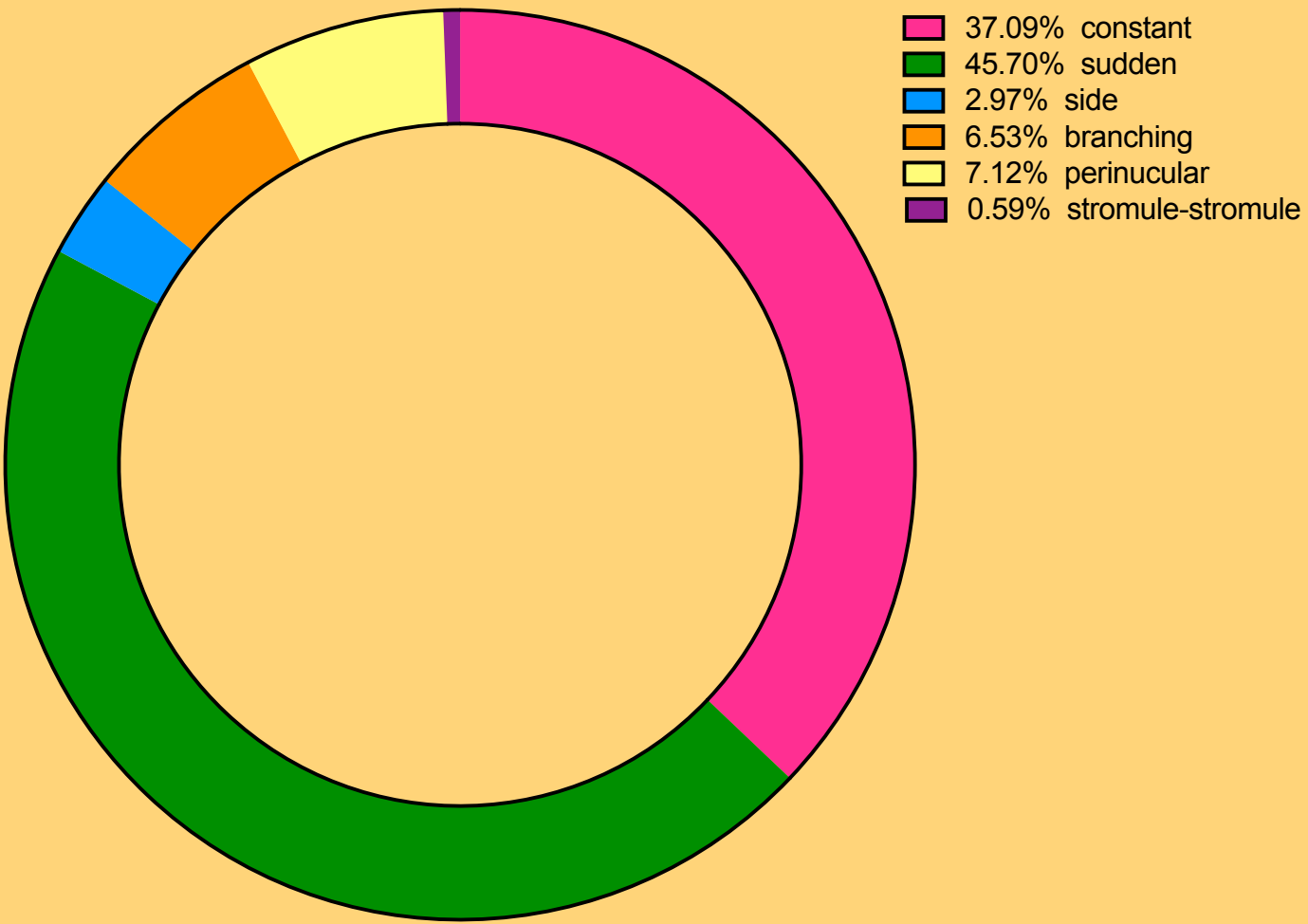

**Total=337**

Supplement: Figure 5—figure supplement 1—source data 1. — The statistics of quantitative analysis of stromule length and movement in VIGS-NbGCP4 plants by Prism7 is included the raw data points, statistical analyses, as well as the original graphs of Figure 5—figure supplement 1A and B. [file elife-23625-fig5-figsupp1-data1.zip › Figure 5-figure supplement 1-source file 1/stromule movement with new data [VIGS-EV].pdf]

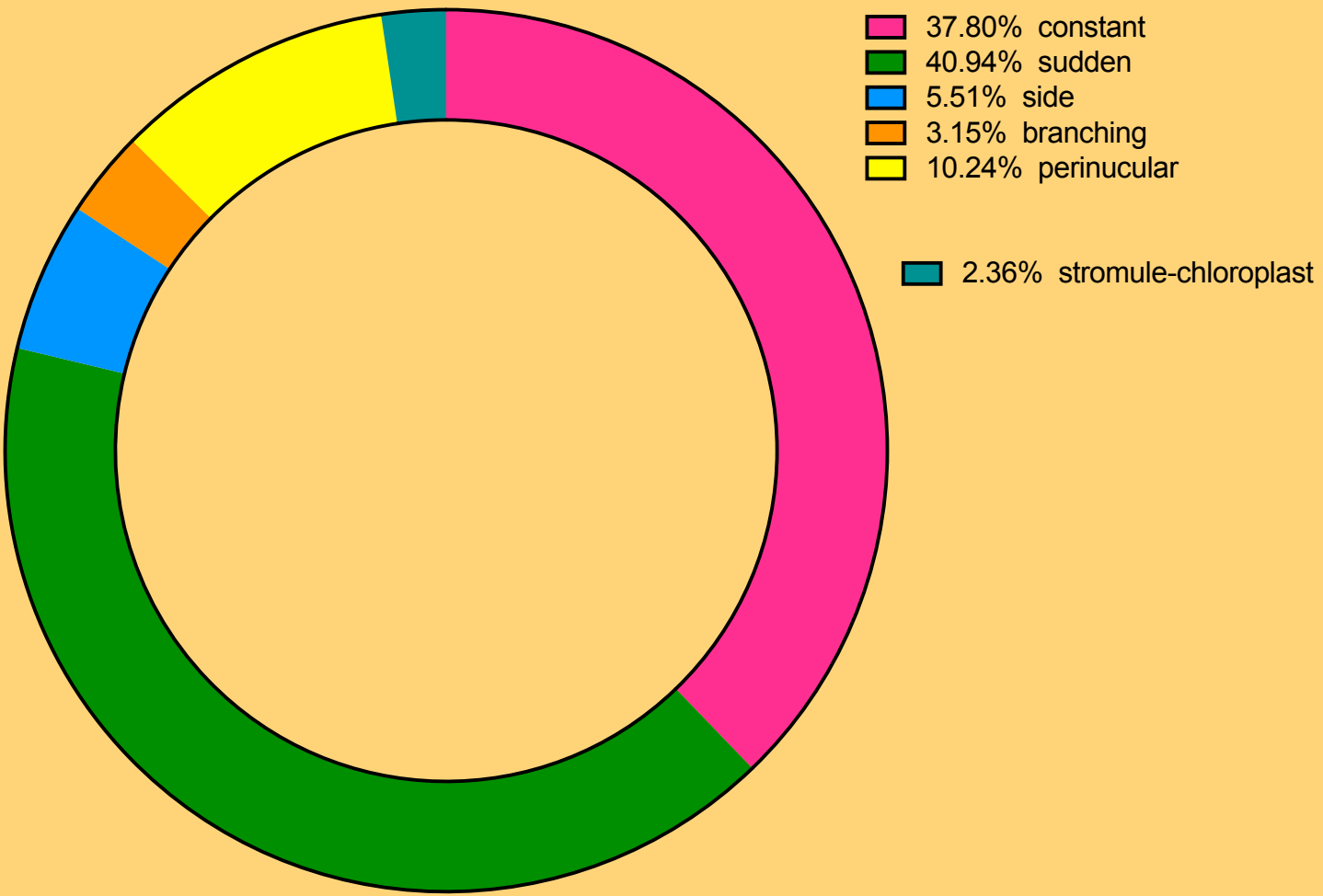

**Total=127**

Supplement: Figure 5—figure supplement 1—source data 1. — The statistics of quantitative analysis of stromule length and movement in VIGS-NbGCP4 plants by Prism7 is included the raw data points, statistical analyses, as well as the original graphs of Figure 5—figure supplement 1A and B. [file elife-23625-fig5-figsupp1-data1.zip › Figure 5-figure supplement 1-source file 1/stromule movement with new data [VIGS-GCP4 +p50].pdf]

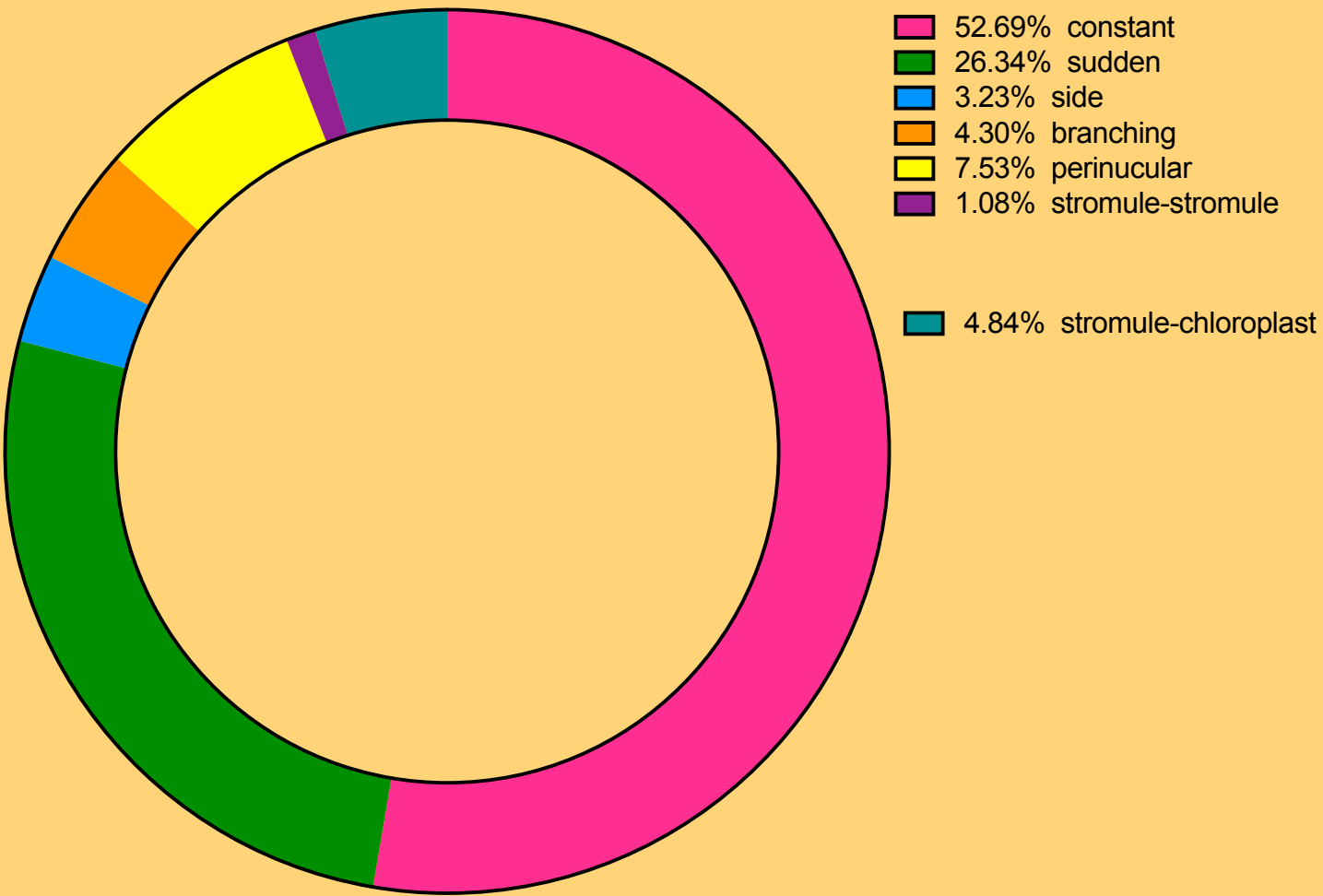

Total=186

Supplement: Figure 5—figure supplement 1—source data 1. — The statistics of quantitative analysis of stromule length and movement in VIGS-NbGCP4 plants by Prism7 is included the raw data points, statistical analyses, as well as the original graphs of Figure 5—figure supplement 1A and B. [file elife-23625-fig5-figsupp1-data1.zip › Figure 5-figure supplement 1-source file 1/stromule movement with new data [VIGS-GCP4].pdf]

**Data 1**

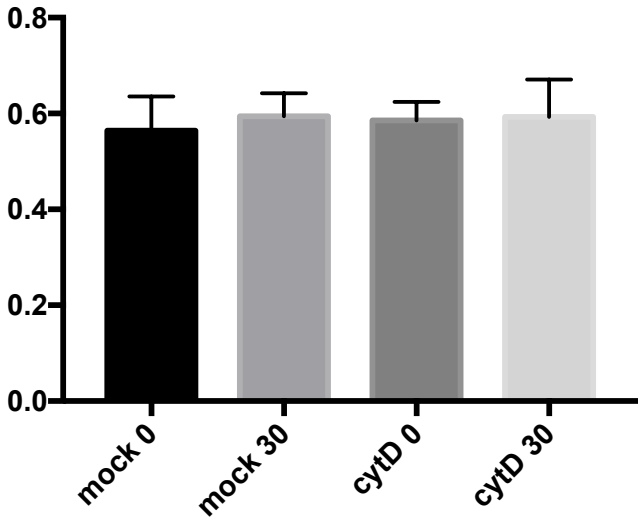

Supplement: Figure 6—figure supplement 3—source data 1. — Statistical analyses and the stromule frequency after CTD treatment to disrupt actin cytoskeleton presented in Figure 6—figure supplement 3B were generated by Prism7. [file elife-23625-fig6-figsupp3-data1.zip › Figure 6-figure supplement 3-source file 1/Data 1.pdf]

# Constant Compare

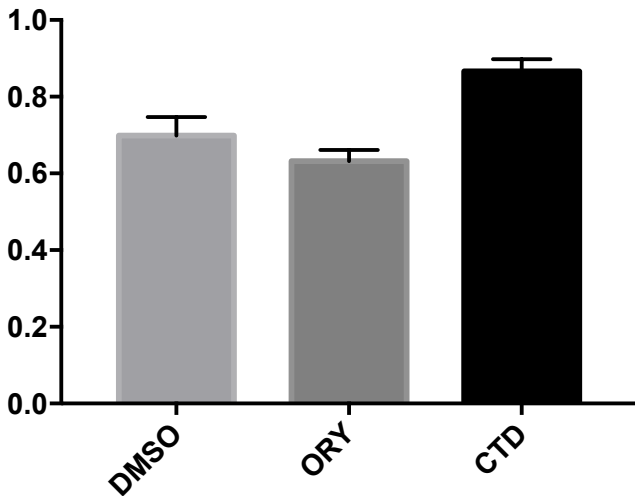

Supplement: Figure 7—source data 1. — All of the statistical analyses and the original graphs of the stromule dynamics presented in Figure 7B-D generated by Prism7 are shown. [file elife-23625-fig7-data1.zip › Figure 7-source file 1/Constant Compare.pdf]

# CTD Chloroplast Movement Type

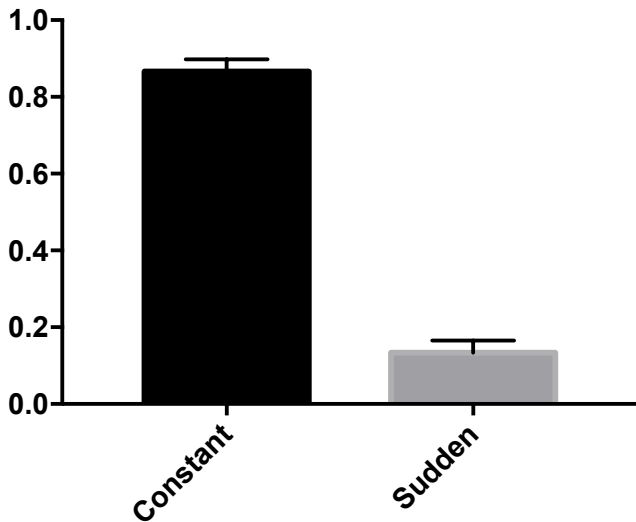

Supplement: Figure 7—source data 1. — All of the statistical analyses and the original graphs of the stromule dynamics presented in Figure 7B-D generated by Prism7 are shown. [file elife-23625-fig7-data1.zip › Figure 7-source file 1/CTD Chloroplast Movment Type.pdf]

# DMSO Chloroplast Movement Type

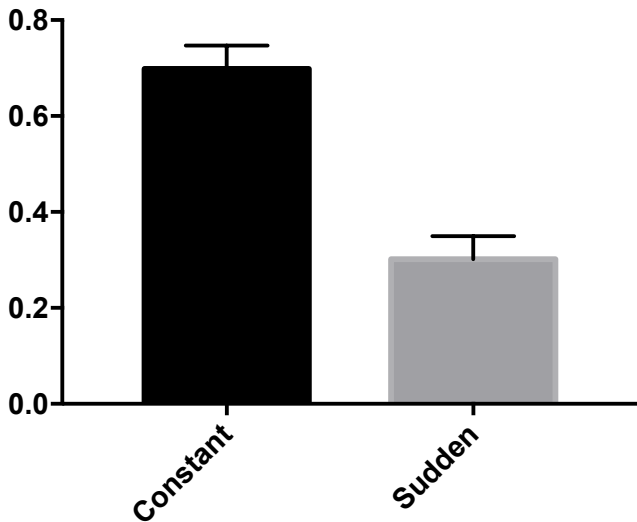

Supplement: Figure 7—source data 1. — All of the statistical analyses and the original graphs of the stromule dynamics presented in Figure 7B-D generated by Prism7 are shown. [file elife-23625-fig7-data1.zip › Figure 7-source file 1/DMSO Chloroplast Movment Type.pdf]

## Extension Velocity-fixed

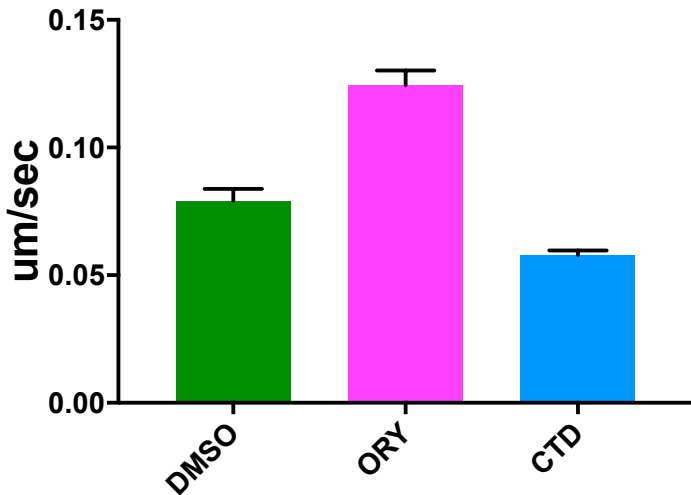

Supplement: Figure 7—source data 1. — All of the statistical analyses and the original graphs of the stromule dynamics presented in Figure 7B-D generated by Prism7 are shown. [file elife-23625-fig7-data1.zip › Figure 7-source file 1/Extension Velocity-fixed.pdf]

# Max Length

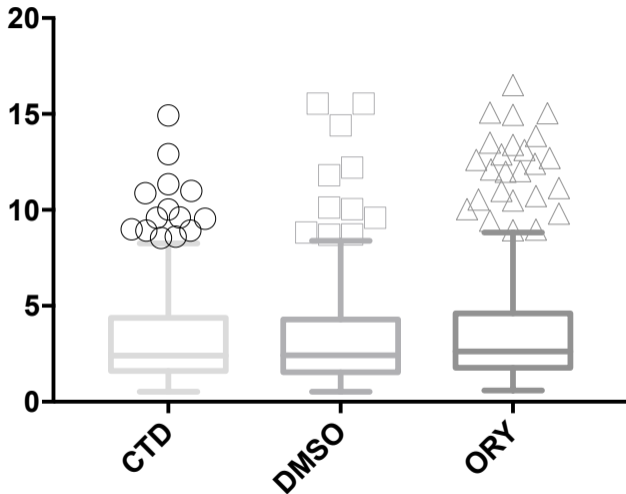

Supplement: Figure 7—source data 1. — All of the statistical analyses and the original graphs of the stromule dynamics presented in Figure 7B-D generated by Prism7 are shown. [file elife-23625-fig7-data1.zip › Figure 7-source file 1/Max Length.pdf]

# Normalized Moving Chloroplasts

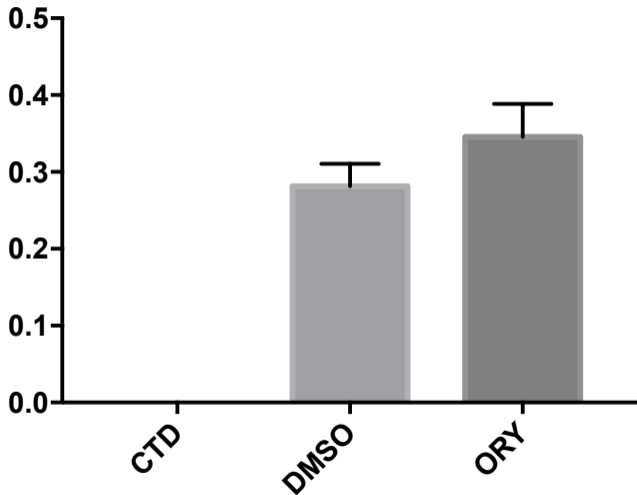

Supplement: Figure 7—source data 1. — All of the statistical analyses and the original graphs of the stromule dynamics presented in Figure 7B-D generated by Prism7 are shown. [file elife-23625-fig7-data1.zip › Figure 7-source file 1/Normalized Moving Chloroplasts.pdf]

# Normalized Stromule Directed Movement

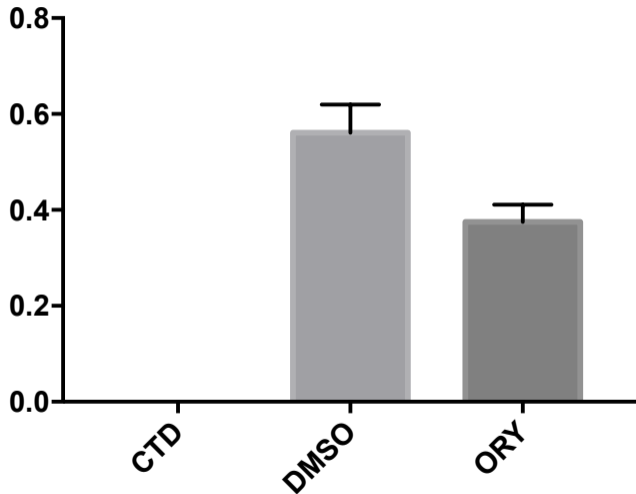

Supplement: Figure 7—source data 1. — All of the statistical analyses and the original graphs of the stromule dynamics presented in Figure 7B-D generated by Prism7 are shown. [file elife-23625-fig7-data1.zip › Figure 7-source file 1/Normalized Stromule Directed Movement.pdf]

# ORY Chloroplast Movment Type

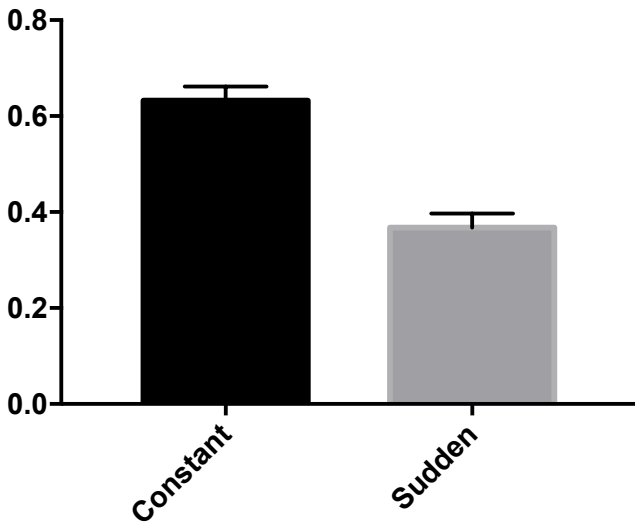

Supplement: Figure 7—source data 1. — All of the statistical analyses and the original graphs of the stromule dynamics presented in Figure 7B-D generated by Prism7 are shown. [file elife-23625-fig7-data1.zip › Figure 7-source file 1/ORY Chloroplast Movment Type.pdf]

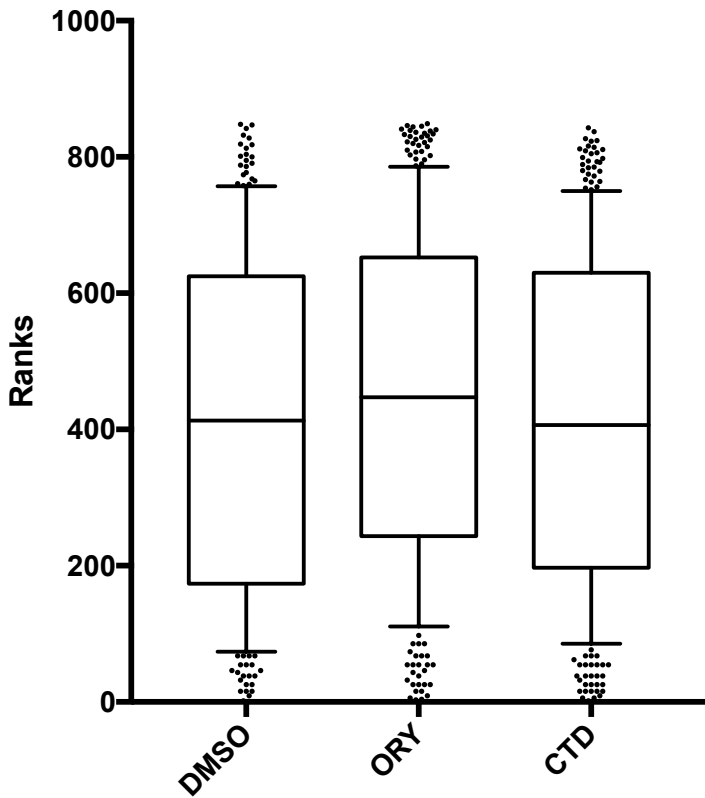

Supplement: Figure 7—source data 1. — All of the statistical analyses and the original graphs of the stromule dynamics presented in Figure 7B-D generated by Prism7 are shown. [file elife-23625-fig7-data1.zip › Figure 7-source file 1/Ranks: Kruskal-Wallis test of Max Length.pdf]

## Retraction Velocity-fixed

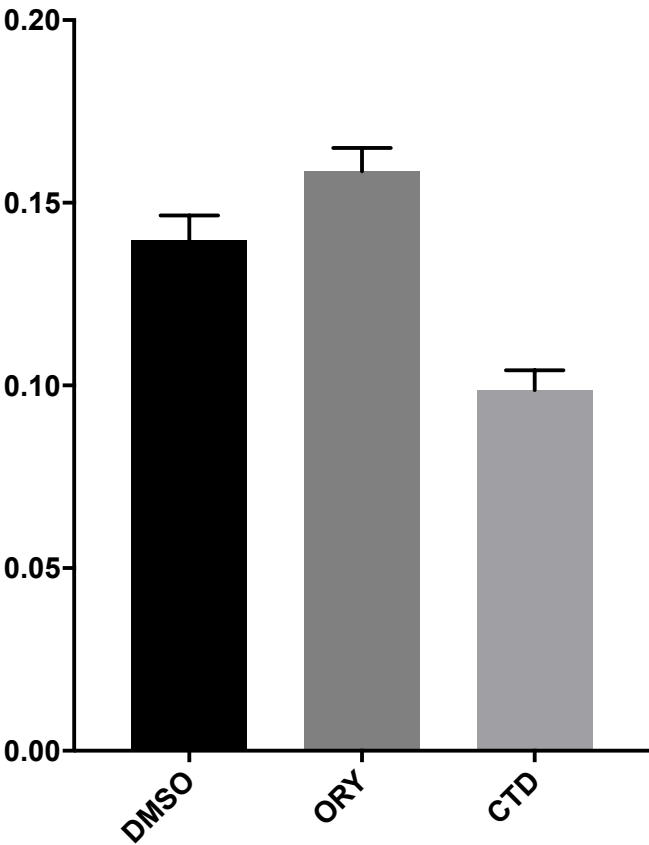

Supplement: Figure 7—source data 1. — All of the statistical analyses and the original graphs of the stromule dynamics presented in Figure 7B-D generated by Prism7 are shown. [file elife-23625-fig7-data1.zip › Figure 7-source file 1/Retraction Velocity-fixed.pdf]

# Sudden Compare

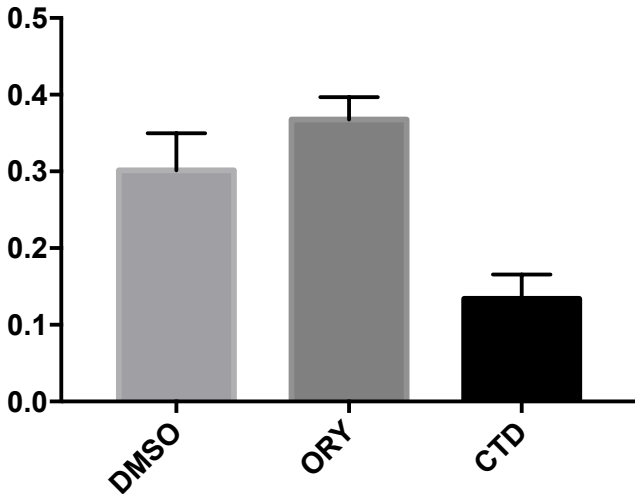

Supplement: Figure 7—source data 1. — All of the statistical analyses and the original graphs of the stromule dynamics presented in Figure 7B-D generated by Prism7 are shown. [file elife-23625-fig7-data1.zip › Figure 7-source file 1/Sudden Compare.pdf]

## 4 and more chl/nucleus

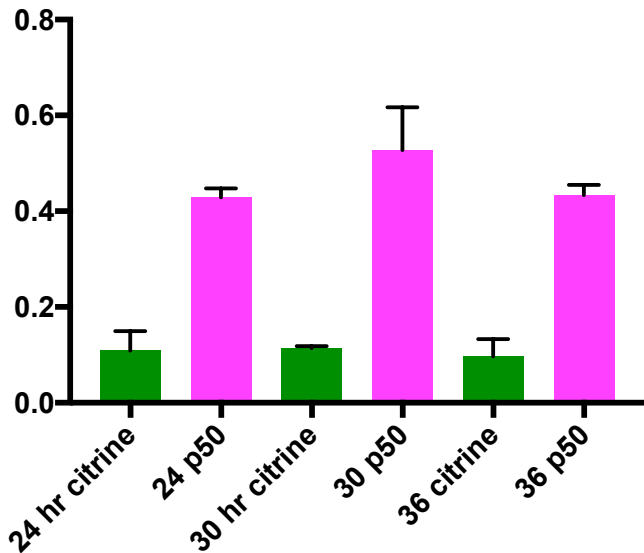

Supplement: Figure 10—source data 1. — Statistical analyses and the graph of the perinuclear clustering of the chloroplasts in TMV-p50 induced plant immune response and presented in Figure 10B and D were generated by Prism7. [file elife-23625-fig10-data1.zip › Figure 10-dource file 1/4 and more chl_nucleus.pdf]

## 4 and mroe p50-drug treatment

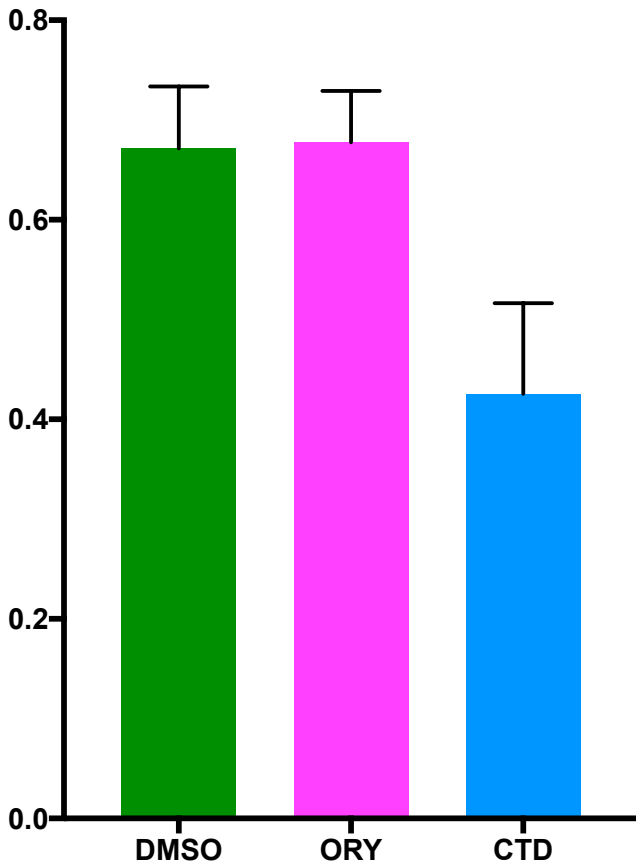

Supplement: Figure 10—source data 1. — Statistical analyses and the graph of the perinuclear clustering of the chloroplasts in TMV-p50 induced plant immune response and presented in Figure 10B and D were generated by Prism7. [file elife-23625-fig10-data1.zip › Figure 10-dource file 1/4 and mroe p50-drug treatment.pdf]
