# Supplementary material for: Stromule extension along microtubules coordinated with actin-mediated anchoring guides perinuclear chloroplast movement during innate immunity
Source: eLife. 2018 Jan 17;7:e23625. doi: 10.7554/eLife.23625 (PMC5815851; doi:10.7554/eLife.23625)
Supplement: Figure 5—figure supplement 1—source data 1. — The statistics of quantitative analysis of stromule length and movement in VIGS-NbGCP4 plants by Prism7 is included the raw data points, statistical analyses, as well as the original graphs of Figure 5—figure supplement 1A and B. [file elife-23625-fig5-figsupp1-data1.zip › Figure 5-figure supplement 1-source file 1/stromule length [GCP4].pdf]

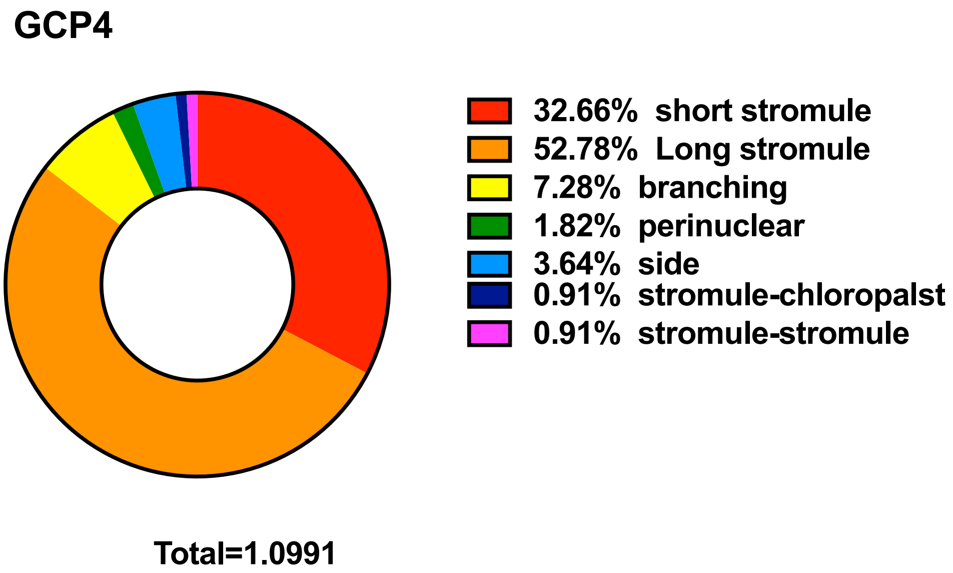
GCP4
32.66% short stromule
52.78% Long stromule
7.28% branching
1.82% perinuclear
3.64% side
0.91% stromule-chloropalst
0.91% stromule-stromule
Total=1.0991
