# Supplementary material for: Stromule extension along microtubules coordinated with actin-mediated anchoring guides perinuclear chloroplast movement during innate immunity
Source: eLife. 2018 Jan 17;7:e23625. doi: 10.7554/eLife.23625 (PMC5815851; doi:10.7554/eLife.23625)
Supplement: Transparent reporting form [file elife-23625-transrepform.pdf]

eLIFE

1st Floor
24 Hills Road
Cambridge CB2 1JP, UK

P 01223 855340
W elifesciences.org
T @elife

eLife's transparent reporting form

We encourage authors to provide detailed information *within their submission* to facilitate the interpretation and replication of experiments. If you have any questions, please contact us: editorial@elifesciences.org.

Sample-size estimation

- You should state whether an appropriate sample size was computed when the study was being designed
- You should state the statistical method of sample size computation and any required assumptions
- If no explicit power analysis was used, you should describe how you decided what sample (replicate) size (number) to use

Please outline where this information can be found within the submission (e.g., page numbers or figure legends), or explain why this information doesn't apply to your submission:

Most experiments in this study are microscopic imaging, which are rather qualitative study than quantitative. Thus, we did not compute our sample size when we designed our experiments. We usually defined one microscopic image as one sample. One image contains more than 100 chloroplasts that we used for calculate stromule number per chloroplast number. In some experiments, these chloroplasts were defined as samples. For example, in figure 4 and Sfigure 3, data from individual chloroplast were used for analyses, resulting in measurement of more than 300 samples for each condition. These are significant sample numbers in most statistics analyses. Thus, computation of sample size is not critical for our study. However, we already know there might be more variation between experiments since stromules are one of the biological responses against stimuli from outside (pathogen infection, in this case). Therefore, we rather increase experimental repeats as technical replicates and add at least two biological repeats. In addition, statistical analyses were done more carefully. We described detail statistics in figure legends and materials and methods.

Replicates

- You should report how often each experiment was performed
- You should include a definition of biological versus technical replication
- The data obtained should be provided and sufficient information should be provided to indicate the number of independent biological and/or technical replicates
- If you encountered any outliers, you should describe how these were handled
- Criteria for exclusion/inclusion of data should be clearly stated
- High-throughput sequence data should be uploaded before submission, with a private link for reviewers provided (these are available from both GEO and ArrayExpress)

eLife Sciences Publications, Ltd is a limited liability non-profit non-stock corporation incorporated in the State of Delaware, USA, with company number 5030732, and is registered in the UK with company number FC030576 and branch number BR015634 at the address 1st Floor, 24 Hills Road, Cambridge CB2 1JP | August 2014

Please outline where this information can be found within the submission (e.g., page numbers or figure legends), or explain why this information doesn't apply to your submission:

In this study, experiments were repeated more than three times. We defined this experimental repeat as a technical repeat. In each experiment, more than two biological repeats are included. A biological repeat means an independent plant. Information for the replicates can be found in each figure legend.

Statistical reporting

- Statistical analysis methods should be described and justified
- Raw data should be presented in figures whenever informative to do so (typically when N per group is less than 10)
- For each experiment, you should identify the statistical tests used, exact values of N, definitions of center, methods of multiple test correction, and dispersion and precision measures (e.g., mean, median, SD, SEM, confidence intervals; and, for the major substantive results, a measure of effect size (e.g., Pearson's r, Cohen's d)
- Report exact p-values wherever possible alongside the summary statistics and 95% confidence intervals. These should be reported for all key questions and not only when the p-value is less than 0.05.

Please outline where this information can be found within the submission (e.g., page numbers or figure legends), or explain why this information doesn't apply to your submission:

We have used Prism 7 for our statistical analyses. For each analysis, we first performed normality test to decide the statistical method. To compare two conditions, we chose Student's t-test with Welch's correction due to unequal variances. If the dataset failed to pass the normality test, we used Mann-Whitney test for rank comparison. For stromule length comparison in Figure 4H, due to a huge difference in measurements across all conditions, we used rank transformation and compared the rank. We described our statistical information as detail as possible in each figure legends (see legends for figure 4, for example). We also have a detail description of statistics used in this study in the material and method section.

(For large datasets, or papers with a very large number of statistical tests, you may upload a single table file with tests, Ns, etc., with reference to page numbers in the manuscript.)

Additional data files ("source data")

- We encourage you to upload relevant additional data files, such as numerical data that are represented as a graph in a figure, or as a summary table
- Where provided, these should be in the most useful format, and they can be uploaded as "Source data" files linked to a main figure or table
- Include model definition files including the full list of parameters used

eLife Sciences Publications, Ltd is a limited liability non-profit non-stock corporation incorporated in the State of Delaware, USA, with company number 5030732, and is registered in the UK with company number FC030576 and branch number BR015634 at the address 1st Floor, 24 Hills Road, Cambridge CB2 1JP | August 2014

- Include code used for data analysis (e.g., R, MatLab)
- Avoid stating that data files are “available upon request”

Please indicate the figures or tables for which source data files have been provided:
